# *Rosmarinus officinalis* Ethanolic Extracts Rescues BV‐2 Cells via Modulating Inflammation and Redox Balance: Comparative Study With Carnosol and Carnosic Acid

**DOI:** 10.1002/cbf.70073

**Published:** 2025-04-11

**Authors:** Hatice Ors, Ebru Alimogullari, Sinem Aslan Erdem, Zubeyir Elmazoglu, Asli F. Ceylan

**Affiliations:** ^1^ Faculty of Medicine, Department of Medical Pharmacology Ankara Yildirim Beyazit University Ankara Turkey; ^2^ Faculty of Medicine, Department of Histology and Embryology Ankara Yildirim Beyazit University Ankara Turkey; ^3^ Faculty of Pharmacy, Department of Pharmacognosy Ankara University Ankara Turkey; ^4^ Faculty of Pharmacy, Department of Pharmacology Ankara Medipol University Ankara Turkey

**Keywords:** apoptosis, inflammation, lipopolysaccharide, microglia, redox modulation, *Rosmarinus officinalis* L.

## Abstract

Neuroinflammation generally refers to an inflammatory response within the central nervous system caused by various pathological insults, including infection, trauma, ischemia, and toxins. As the brain's sentinel immune cell, microglia are tasked as the first responders to infection or tissue injury and initiating an inflammatory response. The perennial shrub plant *Rosmarinus officinalis* L. was reported to possess anti‐inflammatory, anticancer, anti‐nociceptive, antidiabetic, neuroprotective, and antioxidative properties. The present study aimed to investigate the effects of *Rosmarinus officinalis* ethanolic extracts on the lipopolysaccharide (LPS)‐induced neuroinflammation model of BV‐2 cells in comparison to carnosol and carnosic acid, phenolic diterpenes of the plant. Ultrasound‐assisted extraction was used to have ethanolic extract of the plant. LPS was used to induce inflammation in BV‐2 cells. Tumor necrosis alpha (TNF‐α), interleukin 1 beta (IL‐1β) secretion, reactive oxygen species (ROS) production, GSH/GSSG ratio, protein carbonyl level, and caspase‐3 activity were evaluated. Inflammation induced by LPS was reduced by the ethanolic extract. Both carnosol and carnosic acid decreased the TNF‐α and IL‐1β levels as well. The ethanolic extract reduced ROS production and protein carbonylation, and increased GSH/GSSG ratio more effectively compared to the effects of carnosol and carnosic acid. Results depicted that caspase‐3 activity was reduced by the ethanolic extract and this effect was more pronounced compared to carnosol and carnosic acid. The present study indicates the ethanolic extract of *Rosmarinus officinalis* rescues BV‐2 cells from apoptosis via alleviating inflammation and oxidative stress.

## Summary

• The use of rosemary in folk medicine has led to speculation about its potential pharmacological effects.
• Here, for the first time, we proved that the ethanolic extract of *Rosmarinus officinalis* rescues BV‐2 cells via alleviating inflammation and oxidative stress in comparison to the diterpenic compounds of the plant carnosol and carnosic acid.

## 1 Introduction

In recent years, it has been shown that inflammatory processes play a pivotal role in the etiopathology of neurological diseases. The details of immune responses have been elucidated and neuroinflammation has been found to constitute an important component of progressive degenerative loss, mediating the pathogenesis of neurodegenerative diseases [1, 2]. Neuroinflammation generally refers to the inflammatory response that occurs by releasing inflammatory mediators by immune system cells, involving all cells within the Central Nervous System (CNS), including neurons, microglia, astrocytes, and macroglia [1, 3, 4, 5, 6]. Most CNS diseases induce a specific cellular response that involves a series of reactions involving complex interactions between various brain cells [7]. The main purpose of the neuroinflammatory response is to re‐establish homeostasis, initially producing a beneficial effect by eliminating various pathogens, removing cellular debris, and promoting tissue repair [8]. However, when this inflammatory response is not appropriately inactivated, prolonged neuroinflammation is detrimental and can lead to the deterioration of healthy tissue, which can seriously impair physiological functioning [5].

Microglia are innate immune cells of the central nervous system. In the adult brain, microglia are ramified, continuously expanding protrusions to evaluate the surrounding environment and recognize invading pathogens, misfolded proteins, chemokines and cytokines, metabolites, inorganic substances, and changes in the extracellular matrix [9]. Activated microglia undergo a dramatic transformation from its resting branched state to an ameboid morphology [10].

Lipopolysaccharide (LPS), commonly known as endotoxin, is a major component of the outer membrane of gram‐negative bacteria (such as *E. coli*) that induces a pronounced inflammatory response [11]. Cytokines such as IL‐1, IL‐6, and TNF‐α cause an increase in the synthesis of inflammatory mediators such as chemokines, free radicals, and NO [12]. Activation of NF‐κB initiates microglia‐mediated phagocytosis, releasing cytokines and activating molecules essential for the function of the adaptive immune response [13].

*Rosmarinus officinalis* L. (rosemary), commonly known as rosemary, is a plant belonging to the Lamiaceae family, but native to southern Europe and Asia, especially the Mediterranean region. However, it can be found all over the world. The fresh leaves of rosemary are used in the food industry as a food flavoring and preservative because of their antioxidant and antimicrobial potential [14, 15]. In folk medicine, rosemary has been used to treat diseases such as stomach pain, dysmenorrhea, headaches, spasms, rheumatic pain, nervous agitation, epilepsy, and depression. Its use has also been reported in improving memory, modulating neuropathic pain, and treating cognitive disorders [15, 16]. Rosemary contains essential oil, phenolic acids, diterpenoids, triterpenoids, flavonoids, and alkaloids [16, 17]. Rosemary and its constituents have exhibited medicinal and pharmacological effects including antioxidant, anti‐inflammatory, antimicrobial, antiviral, anticarcinogenic, antimutagenic, antinociceptive, and neuroprotective properties [18]. Chemical analysis of different rosemary species has shown that the most potent active constituents are phenolic diterpenes, phenolic acids, and triterpenes, including carnosic acid (CA), rosmarinic acid (RA), carnosol (CAR), rosmanol, betulinic acid, and ursolic acid [19].

LPS in microglia is extensively utilized to create neurodegenerative models by activating many pathways that cause inflammation [11]. Numerous naturally occurring polyphenolic compounds have been shown in earlier studies to have anti‐inflammatory and antioxidant properties to reduce or control neuroinflammation [20]. In this perspective, the present study aimed to investigate the effects of *Rosmarinus officinalis* ethanolic extracts on the lipopolysaccharide‐induced neuroinflammation model of BV‐2 cells in comparison to carnosol and carnosic acid, phenolic diterpenes of the plant.

## 2 Materials and Methods

### 2.1 Chemicals

Carnosol (> 95%) and Carnosic acid (≥ 97%) were purchased from Cayman (Michigan, USA). Dulbecco's Modified Eagle Medium (DMEM) with high glucose and l‐glutamine, Fetal Bovine Serum (FBS) purchased from Capricorn (Ebsdorfergrund, Germany). Trypsin, Penicillin‐Streptomycin and Phosphate Buffered Saline (Dulbecco's PBS 1×) were purchased from Cegrogen (Stadtallendorf, Germany). Dimethyl sulfoxide (DMSO) was purchased from Bioshop (Burlington, Canada). 3‐(4,5‐dimethylthiazol‐2‐yl)2,5‐diphenyl‐tetrazolium bromide (MTT) and Lipopolysaccharide (LPS) purchased from Sigma Aldrich (Missouri, USA). IL‐1β ELISA kit and TNF‐α ELISA kits were purchased from Elabscience (Texas, USA). GSH to GSSG ratio detection assay kit, protein carbonyl assay kit, reactive oxygen species (ROS) detection assay kit, and caspase‐3 activity assay kit purchased from Elabscience (Texas, USA).

### 2.2 Plant Material

The aerial parts of *Rosmarinus officinalis* L. were collected during the flowering stage from (Türkiye) in March 2021 and were identified by Prof. Dr. Hayri Duman (Gazi University, Faculty of Arts and Sciences, Department of Biology). The voucher specimen of the plant is stored in the Herbarium of the Faculty of Pharmacy, Ankara University, Ankara, Turkey (AEF 31015). Plant materials were then air‐dried at room temperature in the shadow. Air‐dried plant materials were ground coarsely in a comminuting mill.

### 2.3 Protocol for Ultrasonic‐Assisted Extraction (UAE) of Rosemary

The UAE method was chosen due to its higher extraction and antioxidant efficiency. For extraction, 30.088 g of ground plant material was weighed and 450 mL of 70% ethanol was added at a ratio of 1:15 for optimum efficiency ratio. In terms of the efficiency of extraction, the time spent in the ultrasonic bath was determined as 1 h in total [21, 22].

The air‐dried plant materials were extracted with 70% alcohol using an ultrasonic bath and a magnetic stirrer. Primary extraction was performed using a 60‐min magnetic stirrer + 60‐min ultrasonic bath, respectively. After the extract was filtered through a clean conical flask with pleated filter paper, 70% ethanol was added to the residue for secondary extraction, and the same extraction procedure was applied again. Then, it was concentrated under vacuum by using a rotary vacuum evaporator (HeivapPresición HL G3 from Heidolph Technologies, Nuremberg, Germany) and dried by lyophilization (IlShin Freeze Dryer from IlShinBioBase, Maxwellstraat, Netherlands). The dried extract was then weighed and stored at +4°C in an amber vial until further analysis.

### 2.4 Chemical Characterization of Rosemary Extract

High performance liquid chromatography (HPLC) was used for the chemical characterization of rosemary extract. Analyses were carried out on an Agilent Technologies 1200 series HPLC (Agilent, Santa Clara, CA, USA), which included a vacuum degasser, binary pump, autosampler, and diode array detector. The analytical method of Choi et al. was used with some modifications [23]. Chromatographic separations were performed on an Eclipse XDB‐C18 (4.6 × 150 mm, 5 μm) column. Separation was performed with a gradient elution at a flow rate of 0.6 mL/min using a mobile phase consisting of a aqueous solution of phosphoric acid (A) and methanol (B).

### 2.5 Cell Culture

BV‐2 microglial cells were kindly provided by Prof. Dr. Cimen Karasu, Gazi University Faculty of Medicine. BV‐2 cell line is derived from C57BL/6 mice brain and has been widely used to create neuroinflammation model due to the difficulty of growing primary neonatal microglial cells (https://doi.org/10.3791/62964). In the present study, cells were cultured in DMEM high glucose complete medium, supplemented with 10% FBS and 1% penicillin‐streptomycin solution at 37°C in a humidified incubator under 5% CO_2_ in T75 flasks. During the culturing period, the medium was changed at intervals of 2–3 days and checked under an inverted‐light microscope until the cells reached 70%–80% confluency. When reached over 70%–80% confluence, cells were seeded onto a 6‐well plate for further experiments.

### 2.6 Cell Treatments

After reaching 70%–80% confluency, BV‐2 cells were seeded in culture dishes and incubated for 24 h at 37°C in a 5% CO_2_ environment. Different dilutions of rosemary extract (2.5–25 µg/mL), CAR (2.5–25 μM), and CA (2.5–25 μM) prepared in 10% DMSO and diluted in medium were applied for 6, 12, and 24 h, and then the viability analysis was performed. For LPS dose determination, LPS (1 mg/mL in PBS) was applied at increasing concentrations (0.05–1.5 µg/mL) diluted in the medium for 6, 12, and 24 h, followed by toxicity analyses. Per the determined concentrations and times, rosemary extract, CAR, and CA were administered to cells simultaneously in the presence of LPS to evaluate the therapeutic effect.

### 2.7 Cell Viability (MTT) Assays

Cell viability analysis was performed based on the traditional MTT (3‐(4,5‐dimethylthiazol‐2‐yl)‐2,5‐diphenyltetrazolium bromide) protocol, which is a colorimetric assay for the evaluation of cell metabolic activity. Briefly, BV‐2 microglial cells were seeded in 96‐well plates and treated with various concentrations of rosemary extract (2.5–25 μg/mL), CAR (2.5–25 μM), CA (2.5–25 μM) and LPS (0.05–1.5 µg/mL) for 6, 12, and 24 h. At the end of the incubation periods, cells were kept at 37°C for 4 h with MTT solution (0.5 mg/mL) prepared in fresh medium. Immediately afterward, the medium was removed and formazan crystals formed were dissolved in 100 μL DMSO (100%). The absorbance values were measured at 570 nm reference wavelengths in a microplate reader (PowerWave XS2; BioTek Instruments Inc., USA). The readings are reported as percent expression of vehicle control groups (*n* = 5). The same procedure was used to evaluate the therapeutic effects. The calculated therapeutic concentrations of rosemary extract, CAR, CA, and LPS were used for the mechanistic studies.

### 2.8 ELISA for Inflammatory Markers

BV‐2 microglial cells were seeded in 96‐well plates and treated with 10 μg/mL rosemary extract, 10 μM CAR, and 10 μM CA in the presence of LPS (1 µg/mL). After 12 h incubation, the levels of IL‐1β (Elabscience, USA) and TNF‐α (Elabscience, USA) in cell culture supernatant were measured according to the manufacturer's instructions using ELISA kits. Results are expressed as percent (%) and represented the mean ± SEM of four experiments in which each sample was examined in duplicate in each experiment.

### 2.9 Reactive Oxygen Species (ROS) Levels

Total intracellular ROS was determined by staining microglia with dichlorofluorescin diacetate (DCFH‐DA). Briefly, cells were incubated with 10 μg/mL Rosemary extract, 10 μM CAR, and 10 μM CA in the presence of LPS (1 µg/mL) in 96 culture dishes. At the end of the incubation period, 20 μM DCFH‐DA prepared in the fresh medium was applied to the cells and kept at 37°C for 30 min. Intracellular production of ROS was measured by the fluorescence detection of dichlorofluorescein (DCF) as the oxidized product of DCF on a microplate reader with an excitation wavelength of 485 nm and emission wavelength of 535 nm. Results are represented by the mean ± SEM of four experiments in which each sample was examined in duplicate in each experiment.

### 2.10 Determination of GSH to GSSG Ratios

Intracellular levels of glutathione (GSH) and oxidized glutathione (GSSG) were determined using the commercial GSH/GSSG assay kit (Elabscience, USA) following the manufacturer's instructions. Cells were incubated with 10 μg/mL Rosemary extract, 10 μM CAR, and 10 μM CA in the presence of LPS (1 µg/mL) in 96 culture dishes. The GSH/GSSG assay protocol uses a proprietary nonfluorescent dye that becomes strongly fluorescent upon reacting with GSH. GSSG levels were calculated by subtracting GSH from total glutathione levels. Results represent the mean ± SEM of four experiments in which each sample was examined in duplicate in each experiment.

### 2.11 Measuring Protein Carbonyl Levels

Intracellular protein carbonyl concentrations were determined using the commercial assay kit (Elabscience, USA). BV‐2 microglial cells were seeded in 96‐well plates and treated with 10 μg/mL Rosemary extract, 10 μM CAR, and 10 μM CA in the presence of LPS (1 µg/mL). The final values were normalized to the actual protein amount determined based on absorbance at 280 nm. Results are represented by the mean ± SEM of four experiments in which each sample was examined in duplicate in each experiment.

### 2.12 Evaluation of Caspase‐3 Activity

Caspase‐3 activity was measured using a Caspase‐3 Colorimetric Assay kit (Elabscience, USA) according to the manufacturer's instructions. Cells were seeded in 96‐well plates and treated with 10 μg/mL Rosemary extract, 10 μM CAR, and 10 μM CA in the presence of LPS (1 µg/mL). Results are represented by the mean ± SEM of four experiments in which each sample was examined in duplicate in each experiment.

### 2.13 Presentation of Findings and Statistical Analysis

All data are expressed as the mean ± SEM of 4–6 independent experiments. Individual differences between the groups were analyzed using one‐way ANOVA analysis of variance. The Tukey and Bonferroni tests were used to analyze the differences between groups, *p* < 0.05 was considered to indicate statistical significance.

## 3 Results

### 3.1 Content of Rosemary Extract

**Figure 1:**
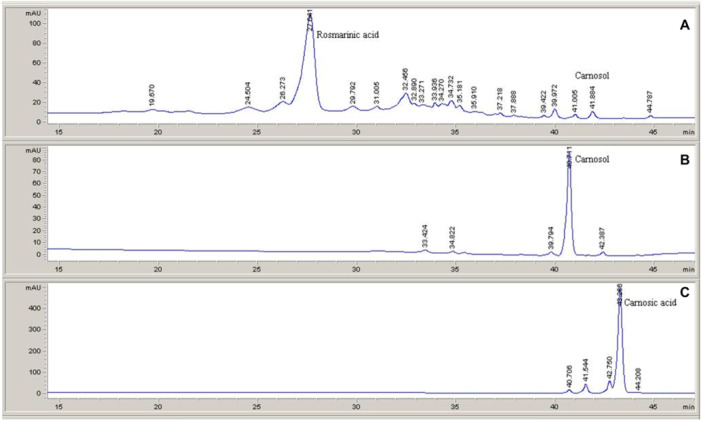
Chromatograms of *Rosmarinus officinalis* ethanolic extract. The chemical characterization of the extract was performed with high performance liquid chromatography (HPLC) based on the method of Choi et al. with some modifications [23]. Rosmarinic acid (A), carnosol (B), and carnosic acid (C).

The analytical HPLC method was optimized to obtain good chromatographic separation of all multiple components of the ethanol extract. Rosmarinic acid, easily identified compared to the standard, is the main component of the phenolic fraction. Carnosol was also detected in the extract, but at a very low level; and carnosic acid was not detected in the extract (Figure 1).

### 3.2 Effects of Rosemary Extract, CAR, and CA on BV‑2 Cell Viability

**Figure 2:**
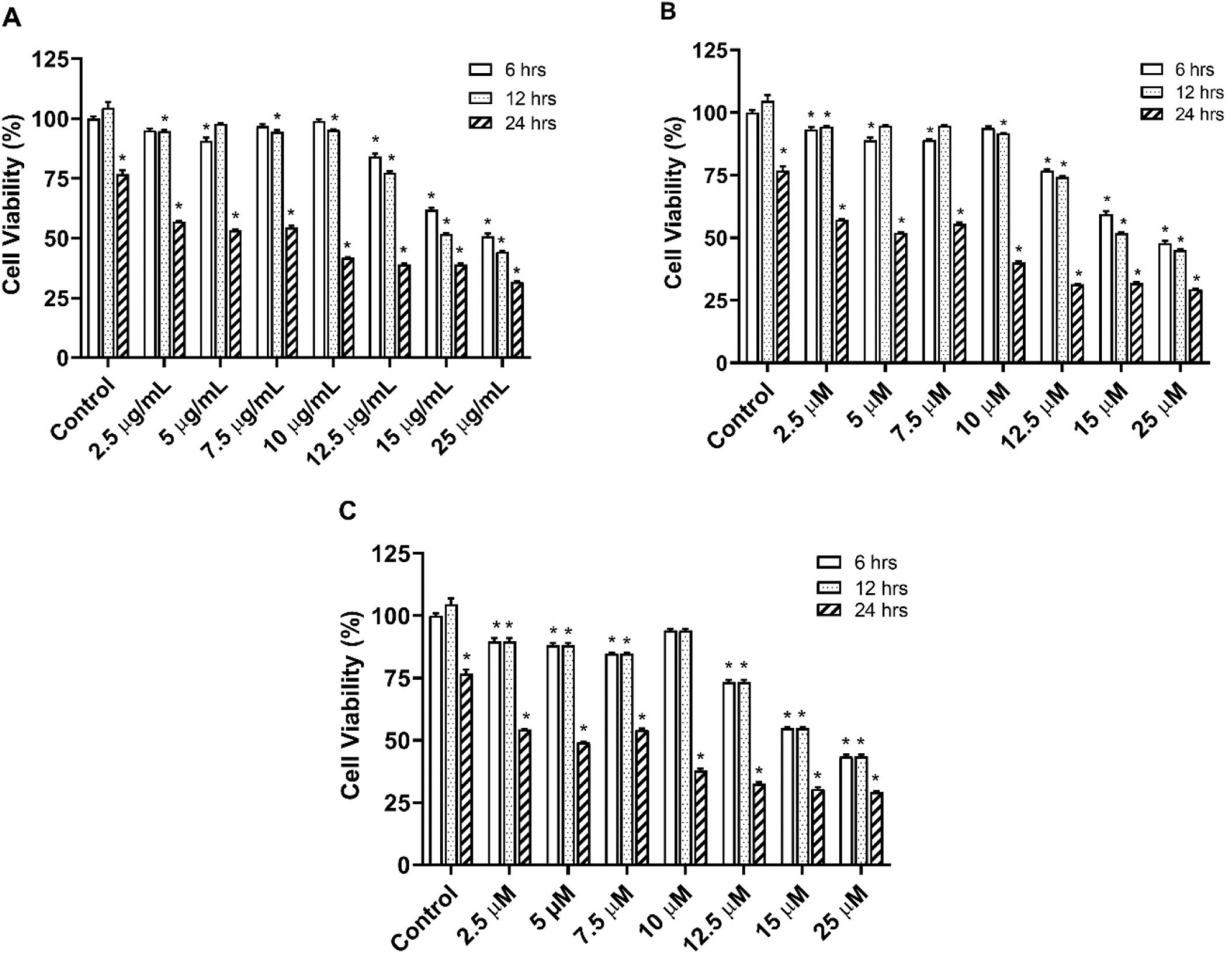
The effect of increased concentrations of *Rosmarinus officinalis* ethanolic extract. BV‐2 cells were treated with either ethanolic extract (A) or carnosol (B) or carnosic acid (C) for 6, 12, or 24 h and the cell viability was detected with MTT assay. All data (*n* = 6) were expressed as mean ± SEM; *p* < 0.05 vs. control (6 h).

After treatment with increased concentrations (2.5–25 µg/mL) of rosemary extract, CAR (2.5–25 M), and CA (2.5–25 M) for different periods (6, 12, or 24 h), BV‐2 cell viability was analyzed with the MTT assay. There was no significant effect in viability with Rosemary extract at 6 h for doses up to 10 µg/mL. There was no significant decrease in viability at doses of 12.5 µg and higher doses (77.49%, 51.73%, and 44.34%, respectively) at 12 h. As for the 24‐h results, a significant decrease in viability was observed at all doses. MTT results with CAR and CA are also similar to those with rosemary extract (Figure 2A–C; *p* < 0.05 vs. control).

### 3.3 BV‐2 Cell Viability Was Increased by Rosemary Extract, CAR, and CA in the Presence of LPS

**Figure 3:**
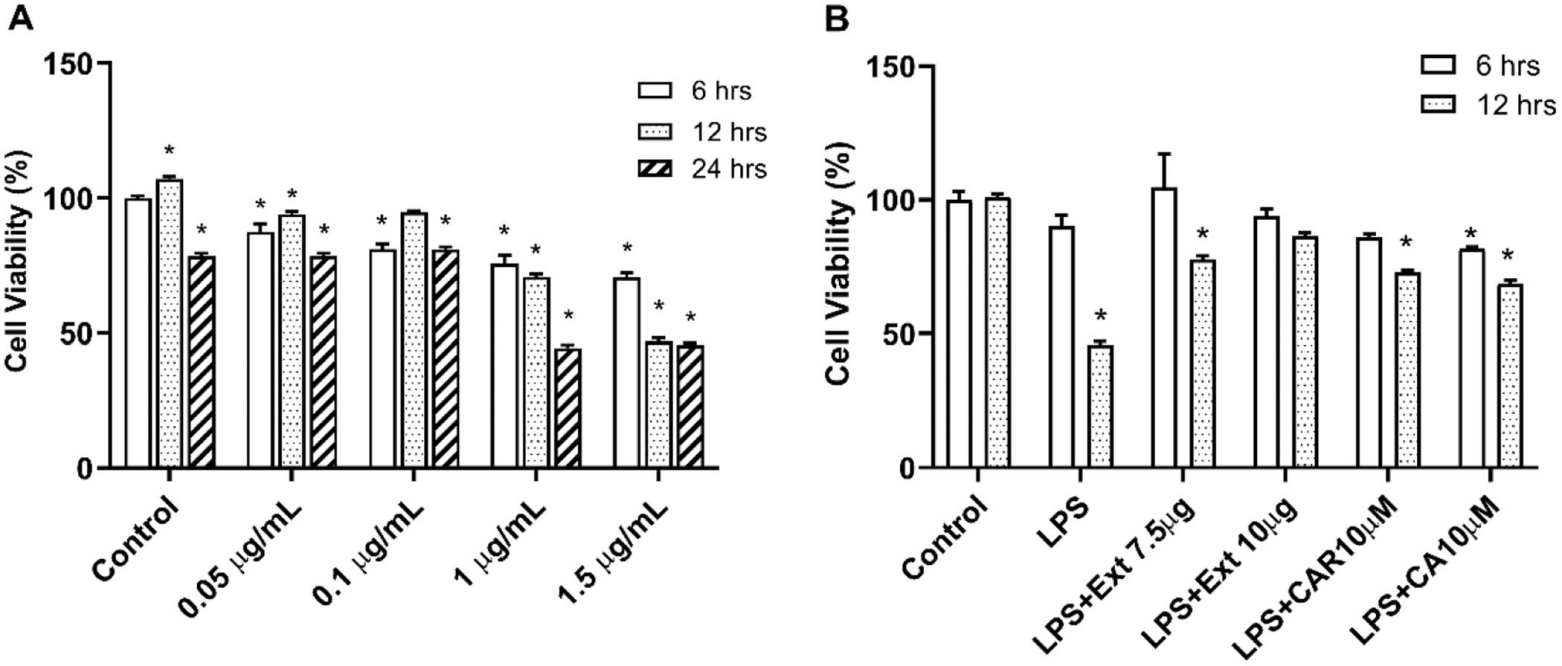
The effect of increased concentrations of lipopolysaccharide (LPS) in BV‐2 cells for 6, 12, and 24 h (A). The effect of *Rosmarinus officinalis* ethanolic extract (Ext), carnosol (CAR), and carnosic acid (CA) in the presence of LPS at 6 and 12 h (B) on BV‐2 cells. MTT assay was performed to investigate the cell viability exposed to the above‐mentioned compounds. All data (*n* = 6) were expressed as the mean ± SEM; *p < 0.05 vs. control (6 h).

For appropriate LPS dose and duration determination, cells were treated with LPS alone for 6, 12, and 12 h. There was no significant decrease in viability at all doses at 6 h of incubation. During the 24‐h incubation, there was a significant decrease in viability at all doses. When all data were considered, a duration of 12 h and a dose of 1 µg/mL were chosen to induce neuroinflammation. To validate these data, a viability assay was conducted with 1 µg/mL LPS administered simultaneously with Rosemary extract or CAR or CA. All these agents ameliorated the decrease in cell viability caused by LPS. However, as shown in Figure 3B, 10 µg/mL extract was more effective than CAR and CA in improving viability.

### 3.4 Oxidative Stress Was Attenuated by Rosemary Extract, CAR, and CA Induced by LPS on BV‐2 Cells

**Figure 4:**
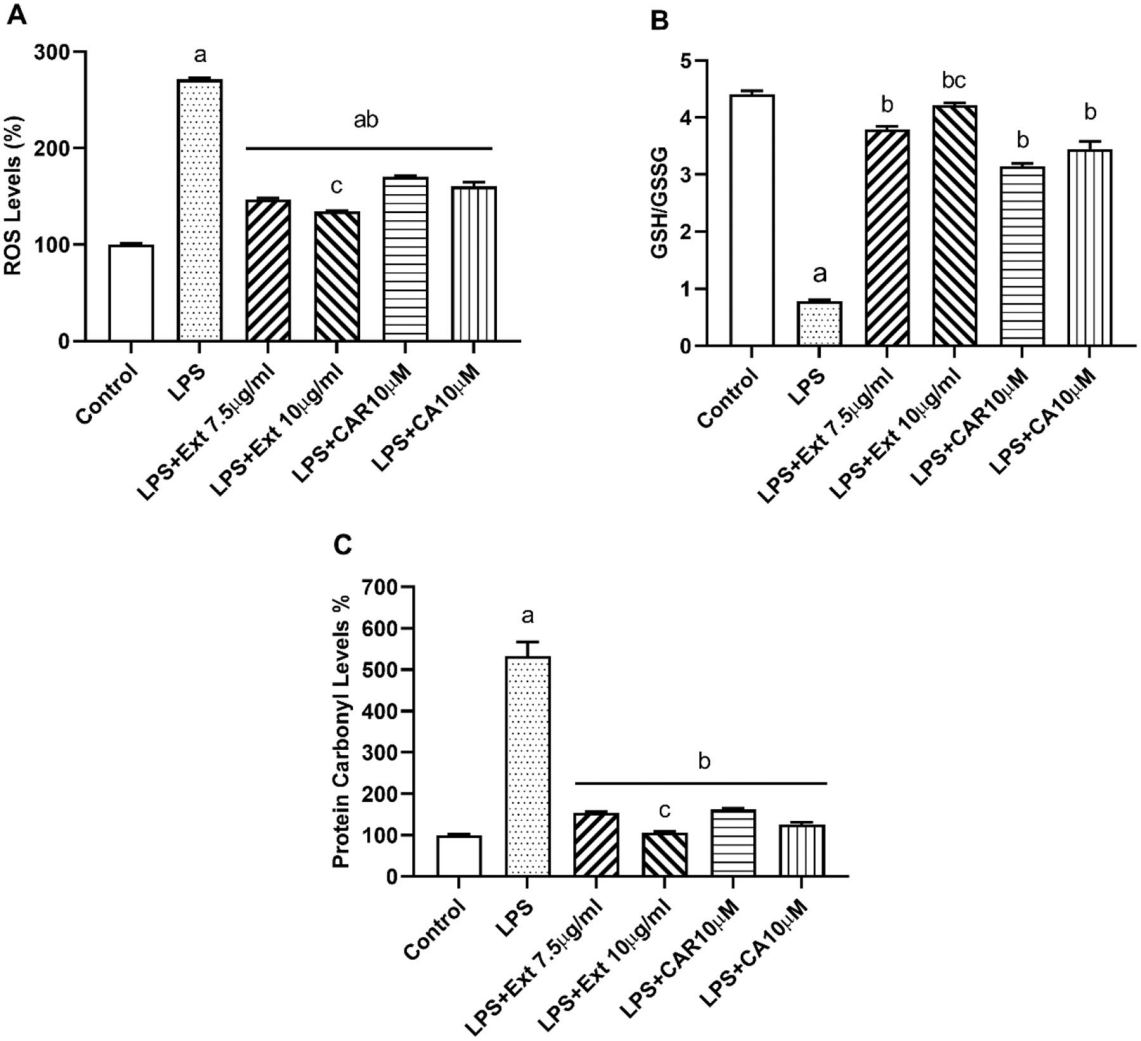
The effect of *Rosmarinus officinalis* ethanolic extract, carnosol and carnosic acid on oxidative stress in the presence of LPS. ROS levels were detected in BV‐2 cells by measuring the fluorescence of dichlorofluorescein (DCF) (A). Protein carbonyl levels were determined with a commercially available assay kit (B). GSH to GSSG ratio were measured with GSH/GSSG assay kit (C) after 12 h of administration. CA, carnosic acid; CAR, carnosol; Ext, *Rosmarinus officinalis* ethanolic extract; LPS, lipopolysaccharide. All data (*n* = 6) are expressed as the mean ± SEM; a: *p* < 0.05 vs. control, b: *p* < 0.05 vs. LPS and c: *p* < 0.05 vs. Ext (7.5 µg/mL).

Activated microglia are known to produce ROS that can cause tissue damage and inflammation. Our results showed that Rosemary extract, CAR, and CA treatment reduced ROS production in the presence of LPS compared to LPS alone. As shown in Figure 3, 7.5 μg/mL Rosemary extract was the most successful in reducing ROS levels (Figure 4A; *p* < 0.05 vs. LPS).

Glutathione, the most abundant antioxidant molecule, is important in the overall cellular redox balance. The GSH/GSSG decreased significantly in LPS‐treated cells compared to control cells. The GSH to GSSG ratio significantly increased with rosemary extract, CAR, and CA in the presence of LPS compared to LPS alone (Figure 4B; *p* < 0.05 vs. LPS).

Oxidation of cellular proteins generates the protein carbonyl groups such as aldehydes and ketones that are related to numerous diseases. As shown in Figure 4C, the protein carbonyl formation was increased by LPS treatment. When cells were treated with the extract, CAR, and CA the formation of protein carbonyl levels was greatly reduced (*p* < 0.05 vs. LPS).

### 3.5 Inflammation and Caspase‐3 Activity Were Reduced by Rosemary Extract, CAR, and CA Induced by LPS on BV‐2 Cells

**Figure 5:**
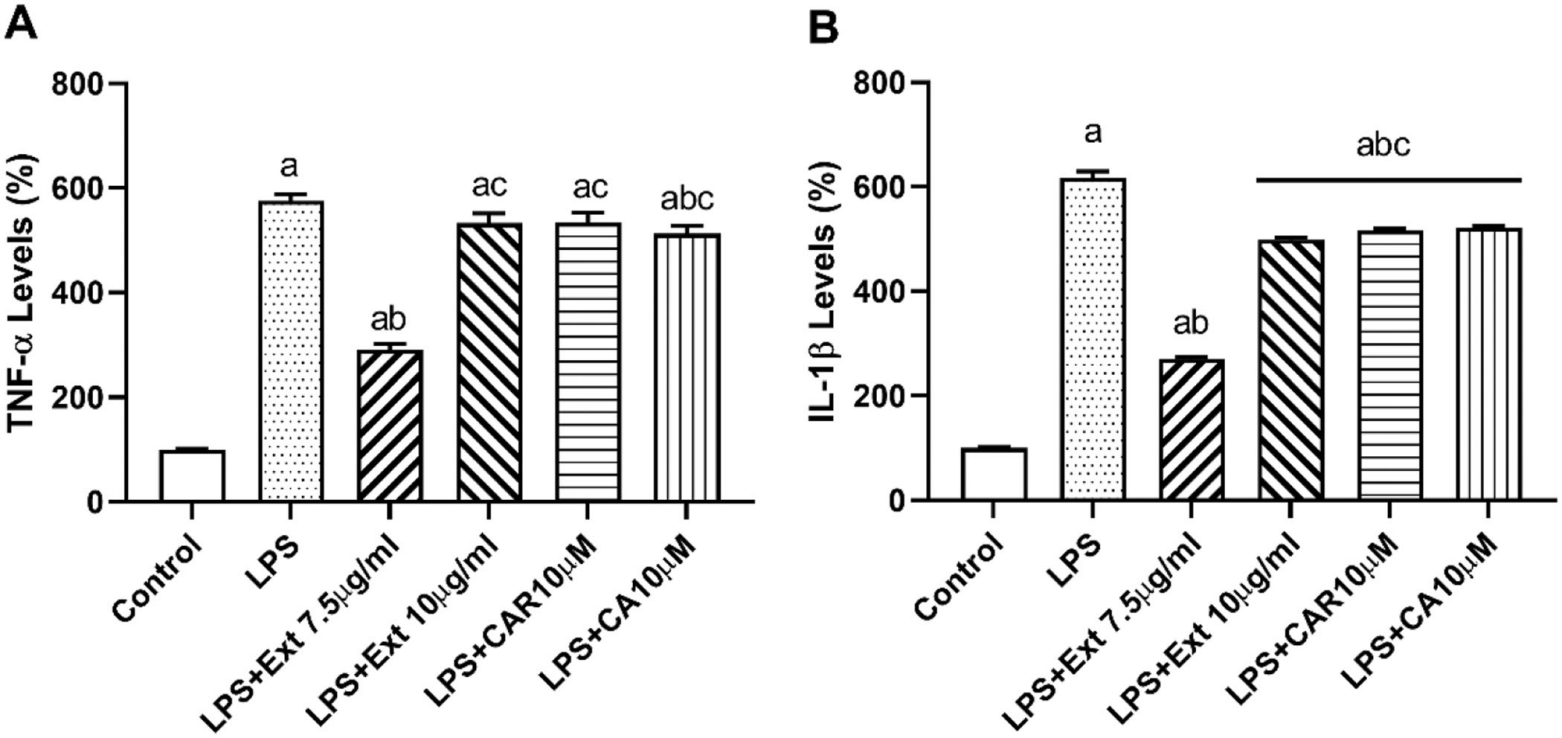
Evaluation of TNF‐α (A) and IL‐1β (B) levels in BV‐2 cells treated with *Rosmarinus officinalis* ethanolic extract, carnosol and carnosic acid after 12 h of administration in the presence of LPS. Both markers were detected with ELISA assay using commercially available kits. CA, carnosic acid; CAR, carnosol; Ext, *Rosmarinus officinalis* ethanolic extract. All data (*n* = 6) are expressed as the mean ± SEM; a: *p* < 0.05 vs. control, b: *p* < 0.05 versus LPS and c: *p* < 0.05 vs. Ext (7.5 µg/mL).

Inflammatory markers (TNF‐α and IL‐1β) increased with LPS (1 µg/mL) treatment (Figure 5A, 5B; *p* < 0.05 vs. control). 7.5 µg/mL extract significantly reduced TNF‐α levels compared to the LPS group, however, 10 µg/mL extract and CA or CAR did not significantly affect onTNF‐α levels. IL‐1β levels increased with 12‐h LPS treatment and decreased with the extract, CAR, and CA. Interestingly, the most effective substance in reducing IL‐1β levels was 7.5 µg/mL extract. The 10 µg/mL extract, CAR, and CA exhibited similar efficiency in decreasing IL‐1β levels (Figure 5; *p* < 0.05 vs. LPS).

**Figure 6:**
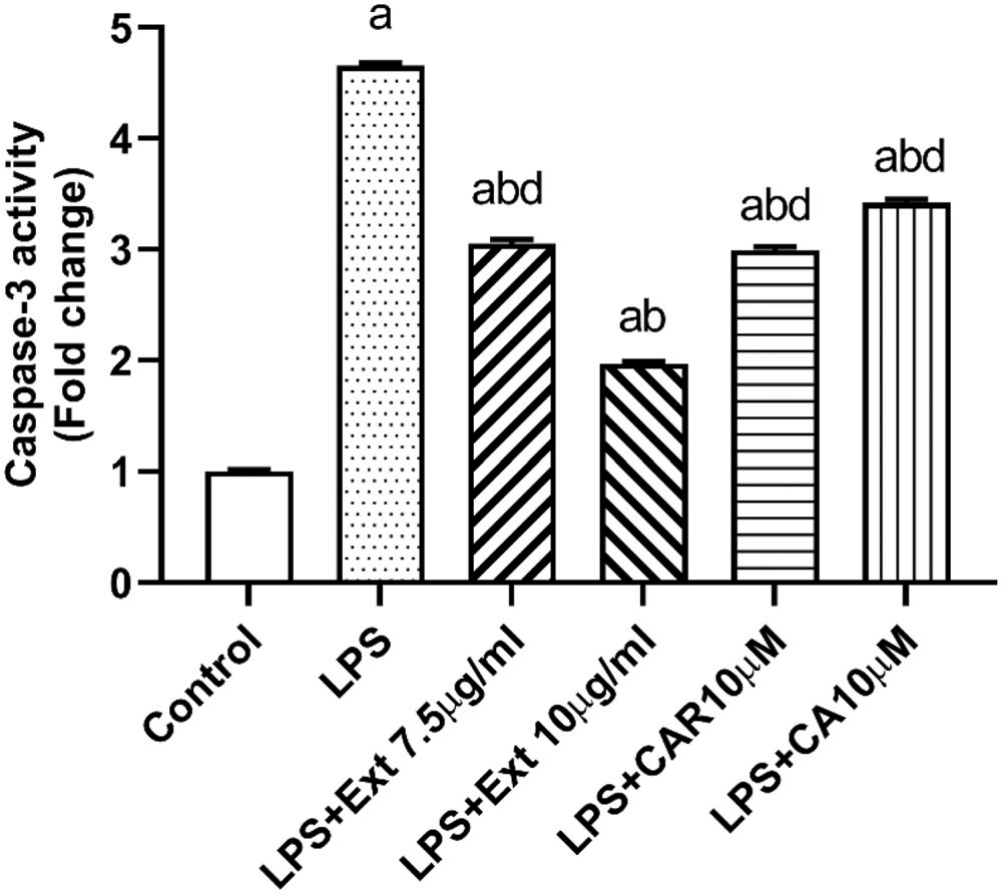
Detection of caspase‐3 activity in BV‐2 cells treated with *Rosmarinus officinalis* ethanolic extract, carnosol, and carnosic acid after 12 h of incubation. The activity of caspase‐3 was determined using a commercially available colorimetric assay kit. CA, carnosic acid; CAR, carnosol; Ext, *Rosmarinus officinalis* ethanolic extract; LPS, lipopolysaccharide. All data (*n* = 6) are expressed as the mean ± SEM; a: *p* < 0.05 vs control, b: *p* < 0.05 vs. LPS and d: *p* < 0.05 vs. Ext (10 µg/mL).

Caspase‐3 an active contributor to apoptosis and inflammation, was significantly reduced by the extract, CAR, and CA compared to the LPS group. In this respect, the most successful outcome was obtained with 10 µg/mL of extract (Figure 6).

## 4 Discussion

In the present study, *Rosmarinus officinalis* ethanolic extract was tested in an LPS‐induced neuroinflammation model on BV‐2 cells in comparison to carnosol and carnosic acid, the phenolic diterpenoids of the plant in the inflammation and oxidative stress perspective.

In recent years, it has been shown that inflammatory processes play a crucial role in the etiopathology of neurological diseases, and neuroinflammation constitutes an important component of progressive degenerative loss, mediating the pathogenesis of neurodegenerative diseases [1, 2]. Uncontrolled activation of microglia by stimuli such as LPS and viral infections can be directly toxic to neurons due to the quantitative release of oxygen‐free radicals and various inflammatory mediators [24]. Therefore, targeting inflammatory pathways may represent a potential therapeutic strategy.

Rosemary has a long history of being associated with memory enhancement. The potential neuroprotective properties suggest that compounds within rosemary may indeed support brain health and cognitive performance. Although the amount of carnosol was very low and rosmarinic acid was found to be the major compound in our extract; carnosol, and carnosic acid have been reported to be the main constituents of the plant [25, 26]. Several environmental factors (e.g., sunlight intensity, water stress), the developmental stage of the plant material when collected, and growth conditions may be responsible for this situation [27].

Rosemary alcoholic extract has been demonstrated to diminish inflammation in neuropathic pain by reducing TNF‐α and IL‐1β [28]. It also shows an anti‐inflammatory effect in LPS‐stimulated J774A.1 murine macrophage cells by inhibiting TNF‐α, IL‐6, NO, and ROS production [14]. Rosemary diterpenes, carnosic acid (CA), and carnosol (CAR) have been shown to reduce LPS‐induced TNF‐α, IL‐1β, and IL‐6, and increase the levels of the oxidized form of glutathione (GSSG) in BV‐2 microglia cells [29]. In the present study, we found that Rosemary extract was more effective than those of CAR and CA in reducing TNF‐α and IL‐1β induced by LPS. LPS‐induced inflammatory response characterized by high levels of oxidant production. Reduction of oxidative stress because of the nature of the terpenic compounds CAR and CAR may have led to reduction of inflammation. The synergistic effect of many phenolic and terpenic compounds in the Rosemary extract may have contributed to more effective results.

Microglial cells are often exposed to high amounts of reactive oxygen species (ROS), and they must possess robust antioxidant defense mechanisms to fend off oxidative damage [30]. GSH (γ‐glutamyl cysteinyl glycine) is significantly expressed in microglial cells. Superoxide anion (O_2_^•^^−^) and other reactive oxygen species (ROS) are scavenged by GSH by direct coupling to oxidation to GSSG or, more quickly, through enzyme‐catalyzed processes. GSH depletion may be a cause or consequence of oxidative stress‐related neurological disorders. In a previous study, astrocytes treated with 50 μg/mL of rosemary extract were able to regain GSH levels and oxidized/reduced glutathione ratio close to control cells [31]. In our study, we found that all substances were significantly effective in reducing ROS levels and increasing GSH to GSSG ratio in microglia. Treatment‐related high GSH levels may be the indication of compound‐mediated ROS scavenging. The ROS scavenging capacity was superior with the extract than those with CAR and CA, and it can be speculated that this superiority may be due to the combined effects of both phenolic and terpenic compounds in the extract.

Protein carbonylation is an important final by‐product of the multiple oxidation pathways occurring in the cell, thus making it a suitable marker of oxidative stress [32]. It has been shown that CAR reduces protein carbonyl levels and ROS [29]. The amount of protein carbonyls in LPS‐stimulated RAW64.7 cells was dramatically enhanced compared to untreated control cells, and when an anti‐inflammatory drug was added, protein carbonyls were about one‐third lower than in LPS‐treated cells [33]. Our results demonstrated LPS‐induced protein carbonyl levels were attenuated by all three treatments. ROS scavenging capacity of the extract, CAR, and CA may be responsible for the amelioration of increased oxidative stress and oxidative stress‐induced protein damage. This beneficial therapeutic effect was more pronounced with the extract.

LPS stimulates microglial cells to activate ROS signaling to release the production of inflammatory markers and induce the apoptotic mechanism in a caspase‐3‐dependent manner. Inhibition of caspase‐3 can prevent neuronal loss and enhance neuroprotection. Therefore, apoptosis inhibitors may be a worthy therapeutic approach in neurodegenerative diseases [34]. CAR and CA inhibited caspase‐3 activity mostly because of the reduction in oxidative stress. Our results indicate the first time that rosemary extract inhibited apoptosis via alleviating oxidative stress and neuroinflammatory responses.

Taking into account all the results gained from the current study, it may be considered that the extract is more effective than those of the main diterpenic substances in reducing inflammation and oxidative stress leading to attenuation of caspase 3 activity in LPS‐induced neurotoxicity. It may be due to the synergistic effect of many phenolic and terpenic compounds in the rosemary extract. It is possible to speculate that the standardized rosemary extract may be an effective therapeutic alternative to modulate neuroinflammation in known neuronal pathologies.

## 5 Conclusion

The present study demonstrates that LPS‐induced neurotoxicity in BV‐2 cells is mediated by inflammation and oxidative stress, which ultimately lead to apoptosis. Importantly, the ethanolic extract of rosemary appears to mitigate these neurotoxic effects by reducing both inflammation and oxidative stress. The data from this study will undoubtedly be instrumental in guiding future research into the in vivo effects of rosemary extract in various animal models of neuroinflammation. While the in vitro findings from the present study are promising, further research is needed to determine the efficacy and safety of rosemary extract in vivo, as the behavior of compounds in cellular models often does not directly correlate with in vivo outcomes.

## Conflicts of Interest

The authors declare no conflicts of interest.

## Data Availability

The data that support the findings of this study are available on request from the corresponding author. The data are not publicly available due to privacy or ethical restrictions.
